# Network Analysis of Drug–target Interactions: A Study on FDA-approved New Molecular Entities Between 2000 to 2015

**DOI:** 10.1038/s41598-017-12061-8

**Published:** 2017-09-25

**Authors:** Hui-Heng Lin, Le-Le Zhang, Ru Yan, Jin-Jian Lu, Yuanjia Hu

**Affiliations:** State Key Laboratory of Quality Research in Chinese Medicine, Institute of Chinese Medical Sciences, University of Macau, Macao, China

## Abstract

The U.S. Food and Drug Administration (FDA) approves new drugs every year. Drug targets are some of the most important interactive molecules for drugs, as they have a significant impact on the therapeutic effects of drugs. In this work, we thoroughly analyzed the data of small molecule drugs approved by the U.S. FDA between 2000 and 2015. Specifically, we focused on seven classes of new molecular entity (NME) classified by the anatomic therapeutic chemical (ATC) classification system. They were NMEs and their corresponding targets for the cardiovascular system, respiratory system, nerve system, general anti-infective systemic, genito-urinary system and sex hormones, alimentary tract and metabolisms, and antineoplastic and immunomodulating agents. To study the drug–target interaction on the systems level, we employed network topological analysis and multipartite network projections. As a result, the drug–target relations of different kinds of drugs were comprehensively characterized and global pictures of drug–target, drug–drug, and target–target interactions were visualized and analyzed from the perspective of network models.

## Introduction

For drugs developed under the target-based drug discovery paradigm, the targets are of critical importance to the drugs since the interactions between the targets and the drugs determines the therapeutic effects of the drugs. The target data of drugs are changeable over time. For example, new targets for existing drugs are continuously being discovered by drug repurposing and repositioning research projects, transforming single-target drugs into multi-target drugs.

In order to identify the changes and trends of drug targets, we retrieved data from Drugs@FDA and DrugBank^1,2^, and analyzed the new molecular entities (NMEs) approved by the U.S. Food and Drug Administration (FDA) between 2000 and 2015 and the relevant target data. Drugs@FDA is a database containing drugs approved by the U.S. FDA, and DrugBank is an integrative and comprehensive database for the drug and relevant drug-interacting biomolecule data including targets, carriers, transporters, and enzymes. Both databases provide abundant source data for our analysis. Furthermore, we employed static and dynamic network analysis approaches to analyze the drug–target relationship.

## Results

### Approved new molecular entities (NMEs) and relevant targets

Over a period of sixteen years, between January 2000 and December 2015, the U.S. FDA approved a total of 361 NMEs. Their 479 corresponding targets were found in DrugBank^2^.

**Figure 1:**
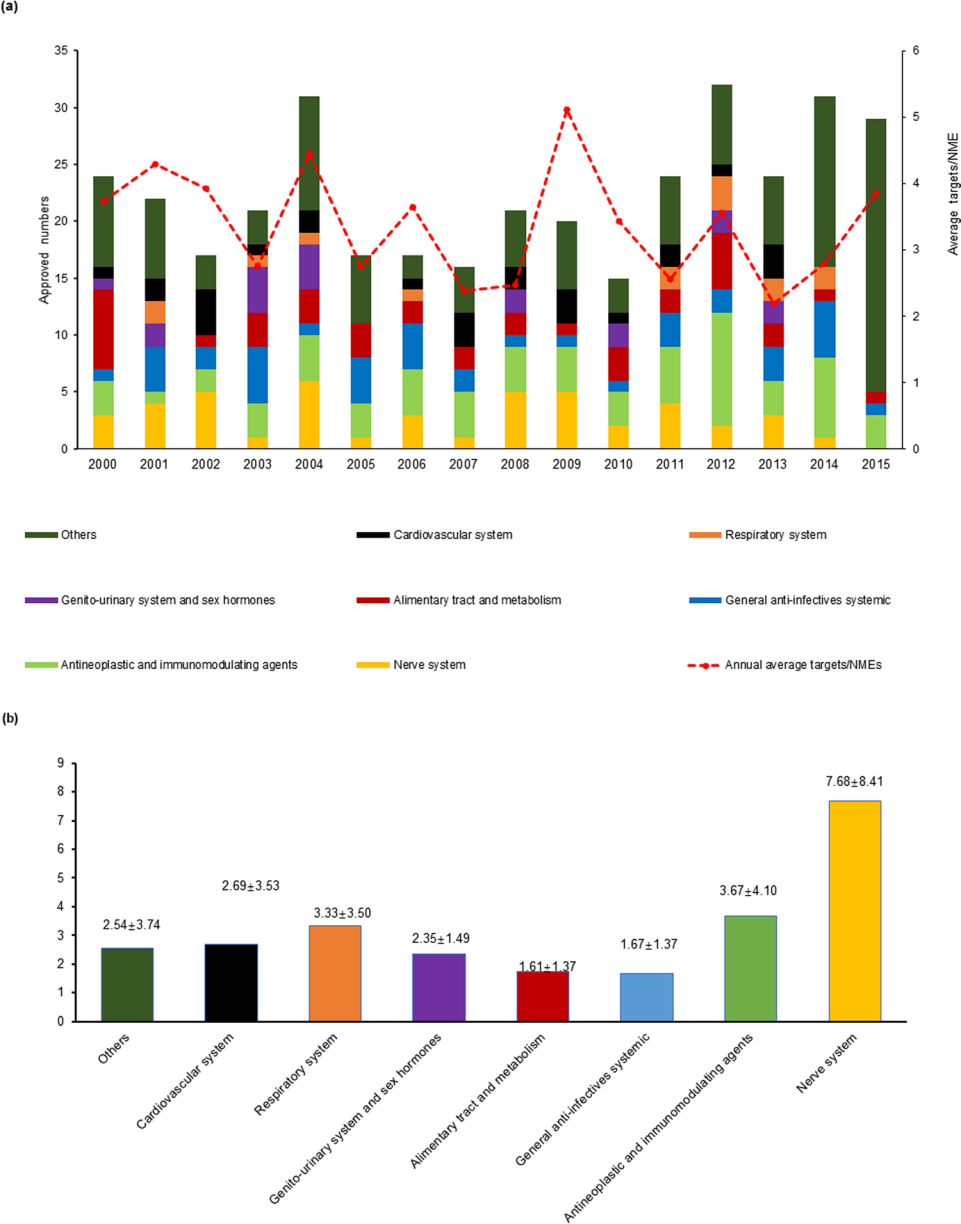
(**a**) Annual number of FDA-approved NMEs between 2000 and 2015 and the average target number of yearly approved NMEs (the folding curve in red). Different types of drugs are colored according to the anatomical therapeutic chemical (ATC) classification system. The average target number of yearly approved NMEs fluctuates around the region of 2 to 5. (**b**) Average target numbers of approved NMEs by ATC since the year 2000. Values above the colored bars indicate the mean ± standard deviation of target numbers.

Three peaks were observed in the histogram of the annual numbers of FDA-approved NMEs. These were in the years 2004, 2012, and 2014, and for each, the number was over 30—more specifically, it was 31, 32, and 31 for 2004, 2012, and 2014, respectively. In 2015 there were 29 NMEs, striking the fourth rank within the 16-year period (Fig. 1a). In the histogram, different colors were assigned to the corresponding types of NMEs approved according to the anatomical therapeutic chemical (ATC) classification system^3^. A glance at the colors of the bar shows that, except for the class of “others”, NMEs of the nerve system (yellow) and antineoplastic and immunomodulating agent classes (light green) have higher ratios than other classes. In addition, the fold line indicating the average target numbers of annually approved NMEs fluctuates between 2.1 and 5.1 (Fig. 1a). NMEs approved in 2013 have the lowest number of average targets per NMEs, whereas the NMEs approved in 2009 are the opposite, making 2009 a fruitful year for multi-target drug approval.

A deeper look into the data of 2013 explains this phenomenon. Of the 24 NMEs approved in 2013, eight of them lack target data and nine have only a single target. Of the remaining seven NMEs, the antineoplastic and immunomodulating agent Dabrafenib Mesylate is the only one with five targets, while each of another two NMEs have three targets, and the remaining four NMEs have only two targets. In contrast, of the 20 NMEs approved in 2009, three lack target data and seven are single-target drugs, whereas the remaining 10 are multi-target drugs. Of these 10 multi-target NMEs, Asenapine (Saphris), Dronedarone (Multaq), Iloperidone (Fanapt), Pazopanib (Votrient), and Milnacipran (Savella) have high target numbers of 20, 18, 11, 10, and 9, respectively. Asenapine, Iloperidone, and Milnacipran are drugs for the nerve system, Dronedarone is a drug for the cardiovascular system, and Pazonib is an antineoplastic and immunomodulating agent.

The neighboring column of the fruitful year of 2009 also interests us. We found that, although 2008 and 2009 have a similar number of approved NMEs, the average number of the targets—2.47 in 2008 and 5.12 in 2009—almost doubles in 2009 (Fig. 1a). A detailed analysis of the data for 2008 indicates that six of the 21 NMEs approved lacked target data and five were single-target drugs. Three of the remaining 10 multi-target NMEs—Tapentadol (Nucynta), Fesoterodine (Toviaz), and Clevidipine (Cleviprex)—have target numbers of six, five and four, respectively. The remaining seven NMEs include three with three targets and four with two targets.

Figure 1b further dissects the distribution of average target numbers by ATC. Upon classification of NMEs into eight classes of ATC, their average target numbers were calculated respectively. As a result, NMEs of the nerve system class enjoy the highest average number of targets. A close look at the distribution of the targets revealed that of the seven NMEs with target numbers greater than or equal to 20, five belong to the nerve system drug class; these are Zonisamide (Zonegran), Ziprasidone (Geodon), Aripiprazole (Abilify), Acamprosate calcium (Campral), and Asenapine with target numbers of 31, 25, 25, 24, and 20, respectively. If the range for observation extends to drugs with target number greater or equal to 11, 20 NMEs are found, of which 12 belong to the nerve system class. These data indicate that many NMEs for the nerve system are not only multi-target, but also have a greater number of targets than NMEs for other classes; therefore, the average target number of nerve system NMEs is greater than that of other classes (Fig. 1b). In contrast, the NMEs of the general anti-infectives class have the lowest average target number (1.38), as most drugs targeting infectious microorganisms are single-target.

### Network analysis of drug–target interactive relationship

In systems biology and network pharmacology, the network is the most frequently employed model for analyzing complex biological systems as it is able to reflect a global picture of the entire system and the interactions/relationships between different components. Hence, based on the drug–target association data available, and through the approaches described in the method section, we modelled two molecular interaction networks—the drug–drug interaction network and the target–target interaction network.

**Figure 2:**
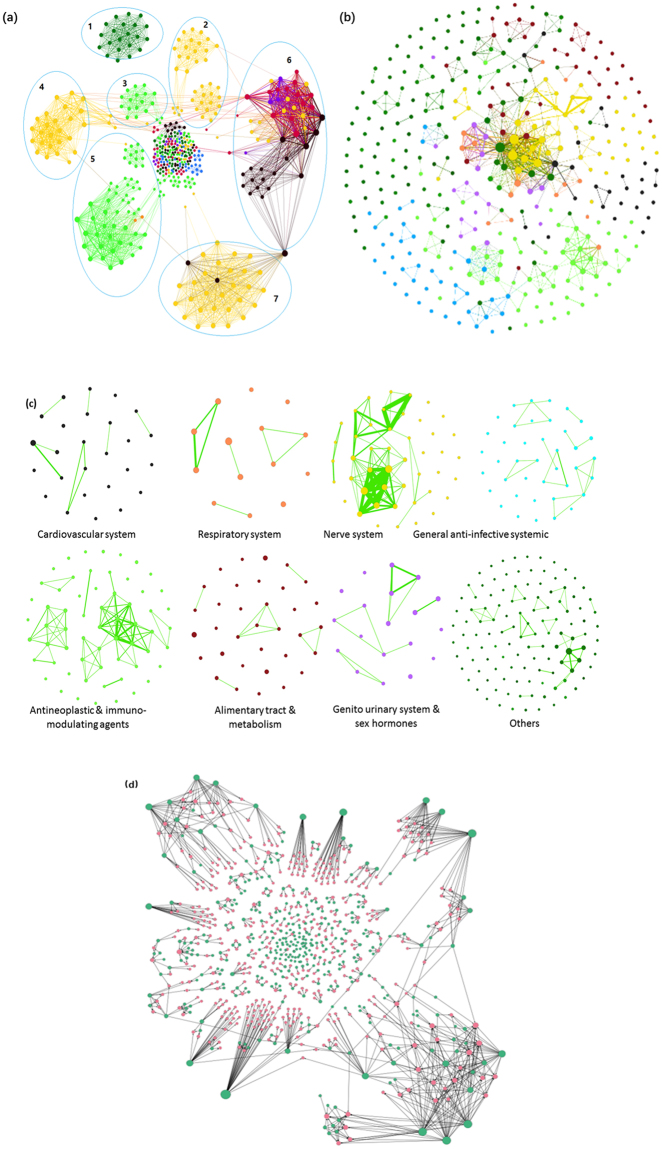
Networks of (**a**) target–target interaction, (**b**) drug–drug interaction, (**c**) drug–drug interaction, and (**d**) drug–target interaction for each ATC class. Nodes with different colors represent targets or NMEs of different ATC codes: respiratory system (orange), alimentary tract and metabolism (red), antineoplastic and immunomodulating agents (light green), cardiovascular system (black), genito-urinary system and sex hormones (purple), general anti-infective systemic (blue), nerve system (yellow), and others (dark green). (**a**) The monopartite target–target network contains 479 nodes and 5,552 edges. Most of the nodes are connected and 63 targets are isolated. Targets of the NMEs from the same ATC category tend to naturally aggregate together into clusters. (**b**) The monopartite drug–drug interaction network derived from the bipartite drug–target interaction network. The drug–drug interaction network consists of 361 NMEs and 656 edges. (**c**) The drug–drug interaction network of each ATC is partitioned to study the drug–drug interactions within each specific therapeutic area. (**d**) The drug–target interaction network. Green nodes are the NMEs, and the red nodes are targets of MNEs.

Upon the construction and visualization of an undirected mono-partite target–target interaction network, we observed several clusters formed by naturally aggregated target nodes. Interestingly, the node colors corresponding to different ATC classes show that, targets of the NMEs from the same ATC code tend to aggregate together into the same cluster. 4 yellow clusters of targets of nervous system NMEs distribute in circles 2, 4, and 7, and the clusters of targets of the antineoplastic and immunomodulating agents are found in circles 5 and 3. At the top of the network is the cluster for targets of NMEs of the “others” class (Fig. 2a, circle 1). Target nodes of NMEs of the alimentary tract and metabolic diseases class (red) and the cardiovascular diseases class (black) form clusters in circle 6. However, in contrast to other clusters, these 2 clusters of different colors connect to each other and appear to be mixed together. Lastly, at the center of the network, many small clusters of targets of different ATC classes’ NMEs aggregate and hence colorful nodes are found. These aggregations are because the edges or links in the projected target–target interaction network are NMEs. The targets of an ATC class’s NMEs have a high probability of being connected by NMEs in the same ATC class. This is why we have aggregated target–target interaction network clusters, which are mostly dominated by a single color.

The structure of networks was also interesting enough for observation and comparison. The overall structure of a drug–drug interaction network (Fig. 2b) is quite different from that of a target–target network (Fig. 2a). In Fig. 2b, large numbers of isolated NME nodes are found in each ATC category because the targets of isolated NMEs are not targeted by other NMEs in the drug–target network (Fig. 2d), while several clusters formed by parts of the interconnected nodes can still be found despite the clusters in the drug–drug interaction network being smaller in size and fewer in number than those in the target–target interaction network. For example, in the central position of the network, there is an obvious cluster dominated by NMEs for the nerve system (yellow), around which other small clusters have formed.

Each ATC category’s NMEs are isolated from the comprehensive drug–drug interaction network. In the drug–drug interaction networks, MNEs are linked via the target. As seen in Fig. 2c, most of the networks are sparse and less tightly connected except those of the nerve system and of the antineoplastic and immunomodulating agents’ drug–drug interaction network. This observation indicates that, in both of the nerve system ATC class and the antineoplastic and immunomodulating agents ATC class, a part of targets are commonly targeted by different NMEs. Specifically, in the nerve system’s drug–drug interaction network, a high number of linkages and links with high weight are observed. This indicates that these two therapeutic areas are very much in need of multi-target therapies.

### Targets of nerve system NMEs have the highest degree in drug–target network

We also detected the high-degree targets in the drug–target interaction network (Fig. 2d). As seen in Table 1, most of the targets with a high degree in the drug–target network are involved in neural processes. Many NMEs are targeting these targets together. Nerve system disorders such as Alzheimer’s disease and Parkinson’s disease are induced by various factors, and multiple receptors are involved^4,5^. Drugs hitting a single target might be insufficient for their treatment. The targets with remarkable aggregation include 5-hydroxytryptamine receptor 3 A, Alpha-2B adrenergic receptor, D(3) dopamine receptor, D(4) dopamine receptor, 5-hydroxytryptamine receptor 2 C, D(1 A) dopamine receptor, muscarinic acetylcholine receptor M4, muscarinic acetylcholine receptor M5, and 5-hydroxytryptamine receptor 1B, and so on (Table 1). These receptors are the targets for nerve system disease treatments. Apomorphine was approved for the treatment of Parkinson’s disease, and the second generation (atypical) antipsychotic drug Aripiprazole was approved for augmentation treatment of major depressive disorders^6,7^; both of them hit more than seven targets. Aripiprazole belongs to the benzisoxazole derivatives; it is a selective monoaminergic antagonist with high affinity for the type 2 5-hydroxytryptamine receptor, dopamine receptors, and histamine H1 receptors. Aripiprazole’s antipsychotic activity is likely to be due to a combination of antagonism at D(2) dopamine receptors in the mesolimbic pathway and 5-hydroxytryptamine 2 A receptors in the frontal cortex.

**Table 1:** The top-ranked targets of the NMEs in the drug–target interaction network.

Target	Degree	Functions	NMEs
5-hydroxytryptamine receptor 3A	9	Regulate transmembrane transport, voltage-gated potassium channel activity, etc.	Aripiprazole, Palonosetron, Netupitant, etc.
Alpha-2B adrenergic receptor	9	Regulate neuron differentiation, neurotransmitter release, MAKP activation, etc.	Aripiprazole, Apomorphine, etc.
D(3) dopamine receptor	9	Regulate cognitive and emotional functioning, synaptic transmission, GPCR signaling, etc.	Aripiprazole, Apomorphine, etc.
D(4) dopamine receptor	9	Regulate synaptic transmission, short-term memory, etc.	Aripiprazole, Apomorphine, etc.
5-hydroxytryptamine receptor 2C	8	Mediate excitatory neurotransmission, synaptic transmission, activation of ERK1/2 cascade, etc.	Aripiprazole, Apomorphine, etc.
D(1A) dopamine receptor	8	Mediate dopamine transport, long-term synaptic depression, GPCR signaling, etc.	Aripiprazole, Apomorphine, etc.
Muscarinic acetylcholine receptor M4	8	Inhibit acetylcholine release in the striatum. Regulate dopaminergic neurotransmission, etc.	Vilanterol, Aripiprazole, etc.
Muscarinic acetylcholine receptor M5	8	Mediate adenylate cyclase inhibition, phosphoinositide degradation, and dopamine transport, etc.	Vilanterol, Aripiprazole, etc.
5-hydroxytryptamine receptor 1B	7	Mediate GPCR signaling, synaptic transmission, affect the central nerve system, etc.	Aripiprazole, Apomorphine, etc.
5-hydroxytryptamine receptor 1D	7	Mediate GPCR signaling, synaptic transmission, affect the central nerve system, etc.	Aripiprazole, Apomorphine, etc.
5-hydroxytryptamine receptor 7	7	Mediate GPCR signaling, synaptic transmission, affect the central nerve system, etc.	Aripiprazole, Epinastine, etc.
Mast/stem cell growth factor receptor Kit	7	Proto-oncogene receptor tyrosine kinase. Activate the Ras/Raf/MEK/MAPK pathway, etc.	Sorafenib, Dasatinib Imatinib, Ponatinib, etc.
Vascular endothelial growth factor receptor 2	7	Regulate embryonic vascular development and angiogenesis, cell migration and proliferation, etc.	Sorafenib, Cabozantinib, Axitinib, etc.

In total, 11 targets with high degree in the network are listed. MEK: mitogen-activated protein kinase kinases; MAPK: mitogen-activated protein kinases; GPCR: G protein-coupled receptor; ERK: extracellular signal-regulated kinases.

Aside from the targets in the nerve system, two targets of antineoplastic and immunomodulating agents—mast/stem cell growth factor receptor Kit (KIT) and vascular endothelial growth factor receptor 2 (VEGFR2)—also showed relative high degree (Table 1). Inhibitors of the tyrosine kinase activities of the two targets have significantly improved the clinical outcomes of patients with relative positive malignancies. In particular, Sorafenib, a small molecule inhibitor simultaneously blocking receptors of KIT, VEGFR2, VEGFR1, platelet-derived growth factor receptors (PDGFR) -α and -β, as well as the pathways of Raf kinases, mitogen-activated protein kinase kinases (MEK), and extracellular signal-regulated kinases (ERK), etc^8^. are thought to be the first molecular target treatment approved for hepatocellular carcinoma therapy. Parts of these kinases are related to cell proliferation and angiogenesis; thus, Sorafenib reduces blood flow to the tumor and improves the overall survival of patients with advanced hepatocellular carcinoma^9,10^.

### Single-target NME versus multi-target NME in number

**Figure 3:**
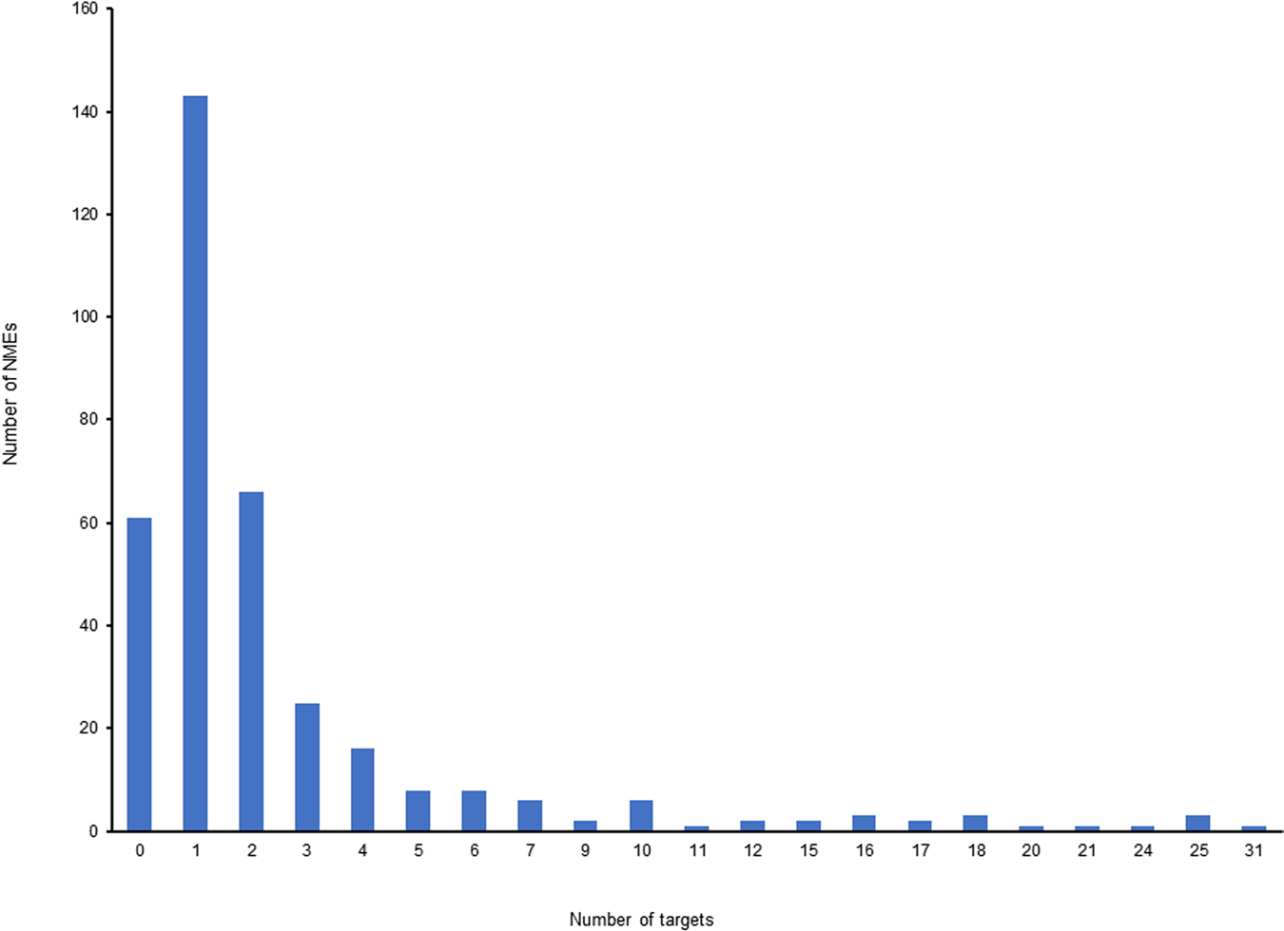
Distribution of NMEs with different target numbers. Single-target NMEs are the most numerous. The value ‘0’ in x-axis refers to NMEs without a known target so far, and there are 61 such NMEs. In contrast, there are only a small number of NMEs with a target number ≥5.

We investigated the distribution of NMEs with different numbers of targets. In all of the NMEs approved by the U.S. FDA between 2000 and 2015, the single-target ones still make up a considerable percentage. Of all 361 NMEs, there are 146 single-target ones, and 61 whose targets thus far remain unknown (Fig. 3). Generally, if an NME has more than one target, it is considered to be multi-target, although in fact the target numbers of multi-target NMEs in this study varies greatly. Besides the single-target NMEs, 66 have two targets, 25 have three, and 16 have four. The remaining 95 NMEs have higher target numbers ranging between 5 and 31. Obviously, the numbers of NMEs with target numbers greater than four are far less than the number of single-target NMEs. This result reasonably matches the average number of 1.33 targets per NMEs in the entire system, with a high standard deviation of ± 4.67 targets, and it also matches the fluctuating fold line of the yearly average target number in Fig. 1a.

### The average nodal degree of networks reflects the trend of multi-target NME development

We also analyzed the topological parameter of networks to mine the features. As explained in the method section, we use the half value of the average nodal degree of the target-target network of different years, i.e., <D>/2, to identify the trends of the multi-target NME development. The average nodal degree of a network is determined by the ratio of the sum of edge number to the sum of the node number, and hence the comparison of average nodal degree value <D>/2, i.e., the ratio of edge number to node number of the target-target network of different years, can reflect the changes of the whole multi-target drug system.

**Figure 4:**
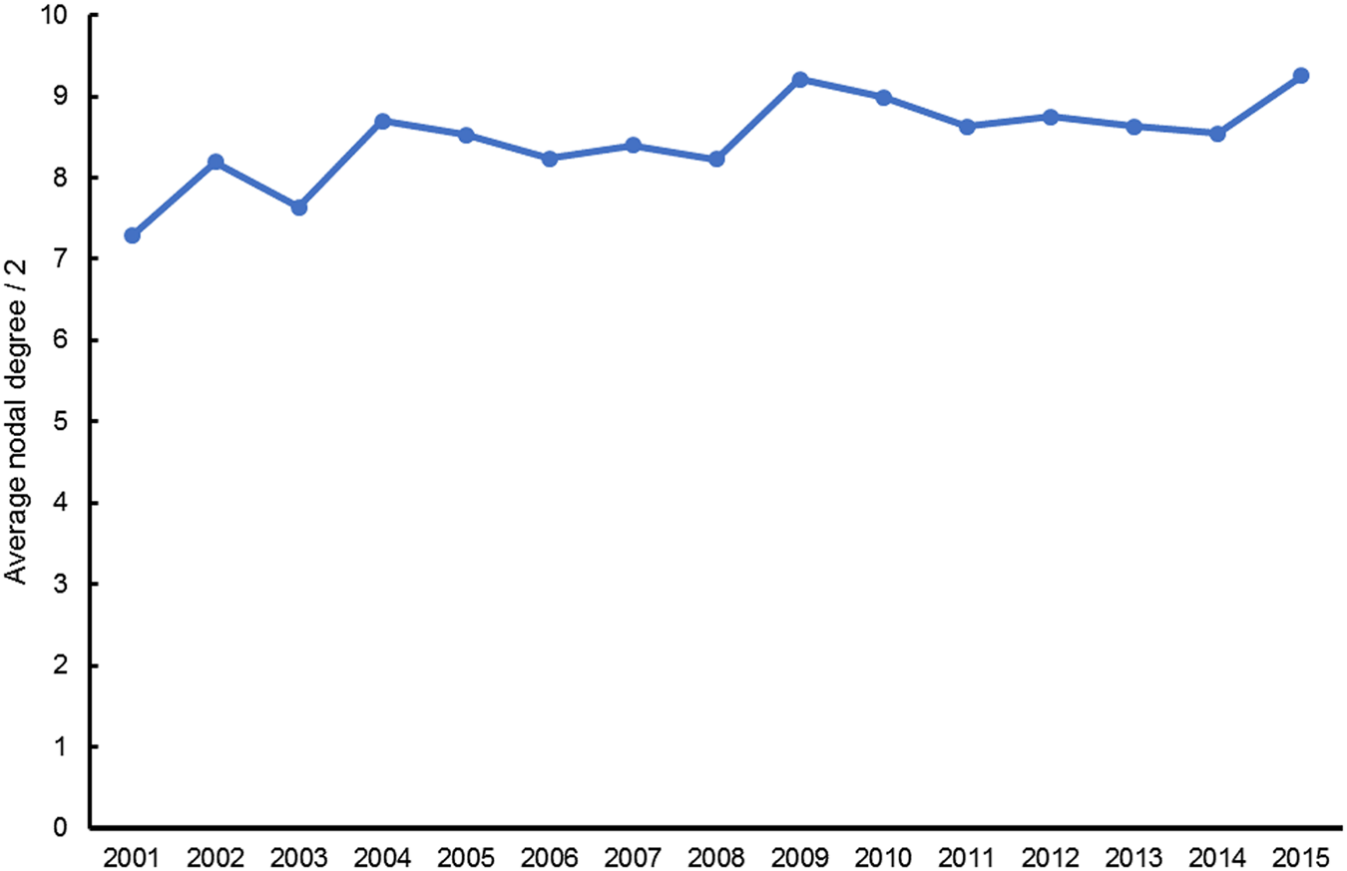
Increasing trend of the <D>/2 value of the target-target networks along the years. It reflects that, the ratio of multi-target NME number to target number are increasing (Note: the average nodal degree <D>/2 value of 2015 refers to the value of the target-target network generated by NME and target data from the year 2000 to 2015 period, instead of the value of the single 2015 year’s target-target network. So does other <D>/2 value of other year numbers in the x-axis).

Our results show an increasing general trend of the <D>/2 value along the years, despite the fluctuations of the folding line (Fig. 4). Every year new NMEs are approved and together with their targets, they are added into and change the topological structure of the target-target network. As mentioned above, the average nodal degree <D> of a network is determined by the ratio of the sum of edge number to the sum of the node number of a network. In the target-target network, an edge stands for a multi-target drug targeting a pair of targets. Therefore, it is indicating the more and more multi-target NMEs are being added into the whole system. What is more, within the 16-year long period from year 2000 to 2015, the increase of the multi-target NMEs are quicker than the target number in general.

## Discussion

Generally, we found two types of the NMEs during the analyses: single-target and multi-target. For a very long time, the drug development paradigm has been focused on and has tightly adhered to the classic “one lock one key” model. Such single-target drugs gained success in many therapeutic areas; however, it is said that the treatment of multi-genic complex diseases, such as neurodegenerative disorders and cancers, require multi-target therapies^11^. Many reports, including our previous work, discuss the shift of the drug discovery paradigm from single-target drug development to multi-target drug development^11–13^. We previously compared the average target number of the NMEs approved by the U.S. FDA between January 2000 and December 2009 with those approved before 2006, and found a slight increase in the average target number of NMEs approved between 2000 and 2009^12^. Based on the results in this study, we found the number multi-target NMEs are increasing, though in terms of the total number, the single-target NMEs approved by the U.S. FDA between 2000 and 2015 dominated (Figs 3 and 4).

In fact, the aims of multi-target therapy could be achieved in another way, i.e. drug combinations. Many studies have demonstrated that the therapeutic effects of combination therapy, sometimes also referred to as cocktail drug therapy, outperform that of monotherapy^14,15^. For example, the pharmacodynamic evidence for the synergism of drug combinations—the additive therapeutic effect—show the advantages of reducing dosage and toxicity, and minimizing drug resistance^16,17^. Hence, the U.S. FDA continues to approve new combinatory usages of existed drugs. In fact, the study of drug combinations is also of high interest.

Whilst, it is also worth noticing that, with the rapid development of molecular and cellular diagnostic techniques and advances in precision medicine, single-target drugs have yielded relative breakthroughs in recent years, particularly in cancer treatment. Newly approved drugs targeting epidermal growth factor receptor (EGFR) and poly ADP-ribose polymerase 1 (PARP1) improved the clinical outcomes of non-small cell lung cancer and ovarian cancer patients^18,19^.

Policies and political factors are significantly affecting the approval of drugs, including the numbers and the types of drug approved. E.g., the U.S. FDA is supporting the approval of orphan drugs in recent years. Therefore, it is necessary to frequently monitor the context of the drug approval rules and the drugs approved.

In summary, the data of NMEs approved by the U.S. FDA between 2000 and 2015 and their targets were analyzed using statistical analysis and network analysis. As a result, we characterized the drug–target relations of different kinds of drugs and the global pictures of the drug–target, drug–drug, and target–target interactions were visualized and modelled into networks. From our results, especially the comparison between single-target NMEs and the multi-target ones, we found the number of multi-target NME is increasing along the years, but still, the amount of single-target NMEs remains to be great. Indeed, we are glad to see the development and approval of more and more multi-target drugs. It is a complementary strategy to single-target drug disocvery.

There are also limitations in this study. First, biologics or biotechnological drugs such as antibodies are playing an increasingly important role in the drug markets and the treatment of complex diseases, particularly cancers. For example, monoclonal antibodies against programmed cell death protein 1 and programmed death-ligand 1 (PD-L1) have good treatment effect on cancers^20,21^. We will consider incorporating these interesting elements into our future work. Second, the categories of ATC studied in the work are also limited. We are also considering extending the scope to the full categories of ATC in a future study. Third, our analysis is based on the target data from DrugBank as of the date that we carried out our analysis. We did not include data from other sources and cannot provide up-to-date results if the target data of DrugBank is updated.

## Methods

### Data

The U.S. FDA approves multiple types of drugs annually, including biologics/antibodies, small molecules, and new drug combinations. In this work, we focus only on NMEs, and thereby exclude other types of drug from the discussion. The data of approved NMEs from January 2000 to December 2015 were retrieved from drugs@FDA^1^. Accordingly, 361 NMEs from drugs@FDA and their 479 corresponding drug targets from the DrugBank database (version 4.3 released on March 3^rd^ 2016) were obtained^2^. Although there are disputes regarding the concept and understanding of drug targets, we use the target data from DrugBank and agree with and adhere to their definition of targets. Note that parts of drugs do not have corresponding target data in the DrugBank.

After the retrieval of the NMEs’ data, they were classified into different categories according to ATC codes. The ATC code/classification is defined by the World Health Organization Collaborating Centre for Drug Statistics Methodology^3^. In total, 14 classes are defined for the classification of the active ingredients of drugs according to the organ or system on which they act and their therapeutic, pharmacological, and chemical properties. In this study, according to the ATC codes, the U.S. FDA approved NMEs downloaded were classified into “7 + 1” classes since we only focus on the former seven classes of the NMEs. Other less relevant classes were classified into the last class named “others”.

### Network construction

In this study, multiple network models were generated from the primary bipartite networks of drug–target interactions. Before introducing the method for constructing networks, we will first give the definitions of drug–target interactions, target–target interactions, and drug–drug interactions in this study. For drug–target interactions, because we retrieved the target data of the drugs from the DrugBank database, we follow the DrugBank’s definition and refer the drug–target interactions to the binding of the drug molecules to the target molecules. In contrast, the definitions of both the drug–drug and target–target interactions are different from that of the drug–target interaction. Instead of the direct binding activities of molecules, the drug–drug and target–target interactions in this study are defined under the context of network analysis rather than the context of physiological or *in vivo* molecular interactions and thus refer to the associated relation between two NMEs or targets.

Upon the manual curation of the source data, the original directed bipartite network models of drug–target interactions was visualized in Gephi^22^. The bipartite drug–target interaction network was further transformed into two undirected monopartite, or so called single mode networks, via a multimode-network transformation algorithm^23^. As a result, nodes in the network of drug–drug interactions are NMEs and the edges become the targets shared by two NMEs. Completely, it should be called the target-mediated drug–drug interaction network, and in this network two drugs are linked by an edge if they share the same target. Similarly, in the drug-mediated target–target interaction network, nodes are the targets and the edges become the NMEs targeting both of the targets.

To be more reader-friendly and to enrich the information on the network graphs, all networks were visually optimized with the following actions. Nodes with a higher degree number were rendered larger node sizes and edges of multiple linkage were set with greater thickness. In drug–drug and the target–target interaction networks, drugs and targets were colored differently according to the classes of ATC.

During the manual data curation, we found the target data of some NMEs was missing from the DrugBank. Two kinds of condition account for this. The first is that the targets of the NMEs are unknown and yet to be discovered. The second is that the administrator of DrugBank has not collected the relevant target data into the database. The missing targets could bring changes to the structure of the networks constructed, which might affect the results to a greater or lesser degree.

### Topological analyses for networks

The topological parameters of the networks, i.e., nodal degree, eccentricity, closeness centrality, and betweenness centrality, were computed using corresponding algorithms^22^.

Specifically, we used the average nodal degree to measure the changes of the target-target interaction network of different period.

$$ < D > =\frac{2E}{N}$$

In the equation above, <D> denotes the average nodal degree of a network. E denotes the sum number of the edge in the network and N denotes for the sum number of the node in the network.

In the target-target interaction network, all the nodes are drug targets and an edge is a drug targeting a pair of targets, i.e., an edge in the target-target network is a multi-target NME. In order to identify the trend of multi-target NME development, we need to detect the time-series ratio of edge number to node number in the target-target network, as the ratio in the target-target network indeed reflects the ratio of NMEs approved so far since year 2000 to NMEs’ targets. Through comparison on the ratio of different years’, we are able to identify the changes and trend of multi-target NME development. Upon the computation of <D>, we plotted the chart of <D>/2 values along the years as <D>’s value in fact comes from the double of sum of edge number in the target-target network (Fig. 4).

### Data availability

The source data for analysis in this work are available in drug@FDA [https://www.accessdata.fda.gov/scripts/cder/daf/] and DrugBank [www.drugbank.ca].

The datasets generated during and/or analyzed during the current study are available from the corresponding author on reasonable request.
